# Frightful Wound of the Skull; Recovery

**Published:** 1869-07

**Authors:** 


*Frightful Wound of the Skull; Recovery.*—Dr. A. C. Folsom in the *Pacific Med. & Surg. Journal,* describes an extraordinary case of recovery from a frightful wound of the skull, resembling the famous tamping-iron case of Dr. Harlow, published in August No. 1868 of this Journal. The accident resulted from a circular saw, the cut extending from the root of the nose to the occipital protuberance, and being nine inches in length in the bones and eleven inches in the scalp, and three inches deep. The bones of the skull fell apart over an inch, and the membranes as well as the substance of the brain were divided. Thirty two minute pieces of bone, together with considerable sawdust were taken from the wound, also a table-spoonful of the substance of the brain, and the saw must have removed as much more. Dr. Folsom saw the case half an hour after the accident and found the pulse 74, full, soft and flowing. The patient was perfectly conscious, free from pain, and the hæmorrhage slight. He thought himself able to walk, and could not tell when the brain, its membranes, or the walls of the cut were touched, even when pressed upon with considerable force. He was sensible when the scalp wound was touched. After removing the hair and cleansing the wound, the edges of the cranial bones were gradually and carefully drawn together with a common tourniquet, the wound in the scalp requiring six stitches. Adhesive plaster completed the dressing. The patient was dismissed after daily attention for three weeks, and at the end of five or six weeks from the date of the injury he resumed his business.

